# Evaluating the interreader agreement and intrareader reproducibility of Visual Field Defects in Thyroid Eye Disease– Compressive Optic Neuropathy

**DOI:** 10.1038/s41433-021-01504-2

**Published:** 2021-04-08

**Authors:** Aylin Garip Kuebler, Kathrin Halfter, Lukas Reznicek, Annemarie Klingenstein, Siegfried Priglinger, Christoph Hintschich

**Affiliations:** 1 Department of Ophthalmology, Ludwig-Maximilians-University, Munich, Germany; 2 Munich Cancer Registry, Institute for Medical Information Processing, Biometry, and Epidemiology, Ludwig-Maximilians-University, Munich, Germany

**Keywords:** Thyroid diseases, Eye manifestations

## Abstract

**Purpose:**

To categorize visual field (VF) defects according to Freitag and Tanking’s (FT) classification in Thyroid Eye Disease-Compressive Optic Neuropathy (TED-CON) and evaluate the interreader agreement and intrareader reproducibility of the classification.

**Subjects and methods:**

In this retrospective, observational study we included medical reports of 96 eyes (51 patients), who underwent VF testing with TED-CON in Ludwig-Maximilians-University (2008–2019). Two readers separately examined the VFs at the time of the TED-CON diagnosis, each offering two readings of the same VF in a time interval of 1 month. None of our patients were diagnosed with only VF testing. The visual field testing was only performed when the inclusion criteria for TED-CON were met.

**Results:**

The most common VF defects upon TED-CON diagnosis were stage 1b defects in FT classification (34.4% for reader 1, 35.4% for reader 2), followed by stage 2b (10.4% for reader 1, 14.6% for reader 2), and stage 3 (10.4% for both readers).

The overall interreader agreement between 2 examiners was substantial for the first reading (69.8% agreement, kappa 0.635 (95% CI [0.525–0.745])) and moderate for the second reading (66.7% agreement, kappa 0.598 (95% CI [0.488–0.708])). The intrareader reproducibility ranged from substantial to almost perfect (78.1% agreement) between readings (kappa 0.736 (95%CI [0.638–0.834])) for reader 1 and 90.6% agreement (kappa 0.885 (95%CI [0.814–0.956])) for reader 2.

**Conclusion:**

We found good BCVA (LogMAR ≤ 0.2), in nearly half of the cases (44 eyes, 45.8%) and also, strikingly near perfect visual acuity (BCVA LogMAR ≤0.1) in 22.9% of the cases (22 eyes) with TED-CON. We conclude that clinicians should be alert to VF defects in the inferior region (stage 1a/1b in the FT classification) even in patients with a good BCVA.

## Introduction

In 1786, Caleb Perry became the first to describe an association between proptosis and hyperthyroidism. In 1835, Graves followed up with the case of a patient with proptosis and prohibited eye closure, also suffering from hyperthyroidism [1]. Nowadays, thyroid eye disease (TED) is considered a common orbital condition, affecting 25–50% of the patients with the Graves’ disease [2]. Although TED is self-limiting, with an active (dynamic) phase lasting up to 24 months followed by an inactive (static) phase, it still has significant impacts on cosmesis, vision and quality of life [3, 4].

Patients with TED can suffer from Compressive Optic Neuropathy (CON), a condition that is challenging to diagnose due to a broad spectrum of potential clinical presentations. In many cases, diagnosing CON requires additional examinations such as tests for colour vision or visual field (VF), orbital imaging, pattern electroretinogram, and/or visual-evoked potentials, some of which depend strongly on the patient’s cooperation. Even with these examinations at hand, diagnosis can be difficult due to multifactorial causes underlying visual loss. Until recently, the lack of standardized diagnostic criteria has remained a major impediment to timely diagnosis and treatment of TED-CON.

A recent article by Freitag and Tanking (FT hereafter) makes an important contribution by offering the first classification scheme for the visual field (VF) defects in progressive TED-CON. This classification, if proven reliable, promises to help clinicians in not only detecting the early signs of VF changes, but also in categorizing the progression of the defects and monitoring their treatment [5].

Our article sets out to use the FT classification to categorize VF defects in 96 eyes diagnosed with TED-CON in Ludwig-Maximilians-University. By comparing categorizations produced by two independent examiners, as well as those produced by the same examiner over multiple readings, we offer the evaluation of interreader agreement and intrareader reproducibility of the FT classification.

## Methods

This observational, retrospective study was conducted in the Department of Ophthalmology, Ludwig-Maximilians-University, Munich, Germany. We obtained ethical approval from the Ethics Committee of the Ludwig-Maximilians-University, Munich, Germany. Our study adheres to the tenets of the Declaration of Helsinki. Our sample includes 51 patients and 96 eyes. Data were compiled and analysed using SPSS Version 25.0 (SPSS Inc, Chicago, IL, USA). We examined the medical charts of all patients with a definitive diagnosis of TED-CON who underwent VF testing in Ludwig-Maximilians-University, Department of Ophthalmology between 2008 and 2019. After collecting the data from the medical charts, we found 116 VFs with a diagnosis of TED-CON. Six VFs were excluded due to high fixation losses exceeding our threshold (malfixation defined as fixation losses ≥33%). Two VFs from both eyes of one patient were also dropped due to the proliferative diabetic retinopathy documented in her medical records, which could affect the VF examination. Also, one eye of a patient with amblyopia and one eye of another patient with a history of retinal detachment were left out. We wanted to have robust data on the VFs and therefore excluded cases with other diseases which could affect the examination results.

Our study set out to apply and evaluate FT’s classification for TED-CON patients [5]. We therefore relied on the same inclusion and exclusion criteria as the authors. All patients in our sample were ≥18 years age and referred to the Ludwig-Maximilians-University, Department of Ophthalmology for the treatment of TED-CON.

The diagnosis of CON was confirmed by one senior ophthalmic plastic consultant (CH). The diagnosis of TED-CON was based on the presence of at least one of the following clinical findings (i) a positive Swinging-Flash-Light-Test (SWFLT), (ii) optic disc oedema, (iii) worsening of best-corrected visual acuity (BCVA) in comparison to the prior visit (≥2 lines), (iv) BCVA with pinhole being ≤0.6 (Decimal); but not due to corneal problems or other pre-existing eye diseases, and/or (v) findings for apical crowding in orbital computerized tomography (CT). The diagnosis was then supported with further examinations (visual field testing, colour vision test by Arden). The method of color vision by Arden is described elsewhere [6].

All patients received a VF examination assessed with the Humphrey Field Analyzer (Carl Zeiss Meditec AG, Jena, Germany) using the Swedish Interactive Threshold Algorithm with 30-2 test pattern and a stimulus of Goldmann size III. The VF testing indicated all patients to have a BCVA with an adequate near correction. Our analysis relied only on the VF tests performed in our facility, which involved one examination completed on each patient/eye to confirm the diagnosis of TED-CON. Our clinic routinely performs additional examinations, such as colour vision by Arden and/or visual field test, to secure the diagnosis of the optic nerve compression.

Exclusion criteria were history of other causes underlying optic nerve pathologies, such as glaucoma, neurologic or vascular optic nerve diseases, corneal opacity, and other relevant eye diseases (e.g., retinal disorders, significant cataract, significant ptosis) documented in patients’ medical records. Three eyes were excluded due to underlying retinal disease (two eyes with diabetic retinopathy, one eye with prior retinal surgery due to retinal detachment).

Similar to FT, we used only the reliable VF examinations in our analysis, where reliability was defined as <33% of fixation losses and <33% of false positive or negative responses [5, 7]. The VF tests were judged by a glaucoma specialist (LR) as well as an ophthalmic plastic specialist (AGK) to determine if they indicated abnormality according the FT classification. The criteria for pathological VF included: (i) having a single point worse than the 0.5% pointwise probability level on the total, or pattern deviation probability plots, and (ii) three clustered points beyond normal limits (*P* < 0.05) and ≥1 point worse than the 1% level on the total or pattern deviation plot [5]. We (LR and AGK) then categorized the pathological cases using the FT classification, which identifies ten parts and three stages to describe the severity of the VF defect. Importantly, each examiner applied the classification twice (randomizing the order) and independently from one another. Comparison of results from the same examiner, and across the two examiners, allowed us to assess intrareader reproducibility, and interreader agreement of the FT classification, respectively.

Summary of the FT Classification [5] for VF defects in progressive TED-CON:

Stage 1 defects are the earliest changes seen in VF in TED-CON patients.

**Stage 1a** is described as a *small inferior paracentral hemifield abnormality* involving 1–4 consecutive points and having at least 1 point at <0.5 or <1%.

**Stage 1b** is described as a *large inferior paracentral hemifield defect* including 5–29 consecutive points, not involving the entire inferior hemifield, with at least one point being <1%.

**Stage 1c** is described as an *inferior altitudinal defect* showing a severe VF loss on the entire inferior hemifield mostly respecting the horizontal midline, with 70% of the points having *p* < 0.5 on pattern deviation plot.

Stage 2 defects can be described in 2 levels of severity and involves an altitudinal VF defect which crosses the horizontal midline and extends to the superior region.

**Stage 2a** is described as an *inferior altitudinal defect with superior advancement* involving the entire hemifield with a superior extension with 1–15 points crossing the horizontal midline nasally, temporally, or both.

**Stage 2b** is described as an *inferior altitudinal plus superior arcuate defect* involving the entire inferior hemifield with a superior extension above the horizontal line in an arcuate form.

**Stage 3** involves *total loss*, defined as a widespread VF loss in 4 quadrants (MD > 20.00 dB).

**Stage X** involves less common VF defects including the following patterns:

- *superior defect* (VF defect in the superior hemifield),
- *central/paracentral* (VF defect predominantly at macular/perimacular region but not contiguous with the blind spot in 15° fixation),
- *enlarged blindspot* (VF defect contiguous with the blindspot), and
- *scatter* (diffuse VF loss in ≥3 quadrants, but also having minimum 1 point at <2% in ≥2 quadrants).

After compiling all the VF records of TED-CON patients according the inclusion criteria, we de-identified the hard-copy reports by blocking out the name and age of the patients, as well as the date of the examination. We randomly assigned each VF report (per eye) a number from 1 to 96. The two readers (LR and AGK) then classified the defects in the de-identified reports according to the ten categories of the FT classification summarized above [5].

The two readers (LR and AGK) never communicated with one another regarding the classification task. Comparing their categorization, therefore, gave us an indication of interreader reliability. In addition, each reader classified the VF records once again, at 4 weeks from the original categorization. At this time, the VF records were still de-identified, and also re-numbered to prevent the reader from relying on his or her recollection. Comparing the two readings within each reader allowed us to assess intrareader reproducibility.

### Statistical analysis

We used the Cohen’s kappa statistic to measure the level of the interreader agreement between AGK and LR [7]. The statistic compares the level of agreement across measurements (so-called ‘observed agreement’) to the level of agreement to be expected by chance alone (known as ‘expected agreement’). As an alternative, we also computed the proportion of agreements between observers within each category of VF. After calculating the kappa values for the interpretation of the agreement we relied on the publication of Viera et al., which was described as kappa value <0 less than chance agreement; 0.01–0.20 slight agreement; 0.21–0.40 fair agreement; 0.41–0.60 moderate agreement; 0.61–0.80 substantial agreement; 0.81–0.99 almost perfect agreement [7].

We used Student’s *t* test to compare numerical variables between individual subgroups. We relied on Pearson’s correlation coefficient to determine bivariate correlations of continuous variables. In all comparisons, we considered a two-sided *p* value of 0.05 or less to be statistically significant. We conducted all analyses with IBM SPSS Statistics version 25.

## Results

This retrospective, single-centre study includes 96 eyes of 51 patients. Thirty-five patients were female, 16 patients were male. Forty-five patients had a bilateral involvement and 6 patients had a unilateral involvement. The mean ± standard deviation (SD) age of the patients was 58.3 ± 10.3 years (range 34–79 years at baseline). On average, fT3 was 2.97 (pmol/litre) (range 0.90–5.80), fT4 was 3.25 (pmol/litre) (range 0.50–22.80), TSH was 3.40 mU/litre (range 0–34.00), TSH receptor autoantibodies (TSH-R) was 18.74 (IU/litre) (range 0.71–110.00). Twenty-one patients were euthyroid, 13 patients were hyperthyroid, and 5 patients were hypothyroid at the time of the first diagnosis. The mean intraocular pressure was 18.2 mmHG (range 12–32). The mean clinical activity score (CAS) was 5.6 (range 1–8).

The diplopia was graded subjectively according to the European Group on Graves’ orbitopathy (EUGOGO) recommendations with a subjective diplopia score (0 = no diplopia; 1 = intermittent, i.e. diplopia in primary position of gaze, when tired or when first awakening; 2 = inconstant, i.e. diplopia at extremes of gaze; 3 = constant, i.e., continuous diplopia in primary or reading position) [8]. Eighteen patients had no diplopia, 12 patients had intermittent diplopia, 8 patients had inconstant (gaze-dependent) diplopia, and 11 patients had constant diplopia. Two patients had missing information on diplopia in their medical charts (that could not be recovered given the retrospective design of the study).

Two independent examiners (AGK and LR) reviewed all the VFs that met the inclusion criteria and classified each VF twice (with examinations at least a month apart, reports de-identified and re-numbered each time) into the 10 categories identified by the FT classification.

**Fig. 1:**
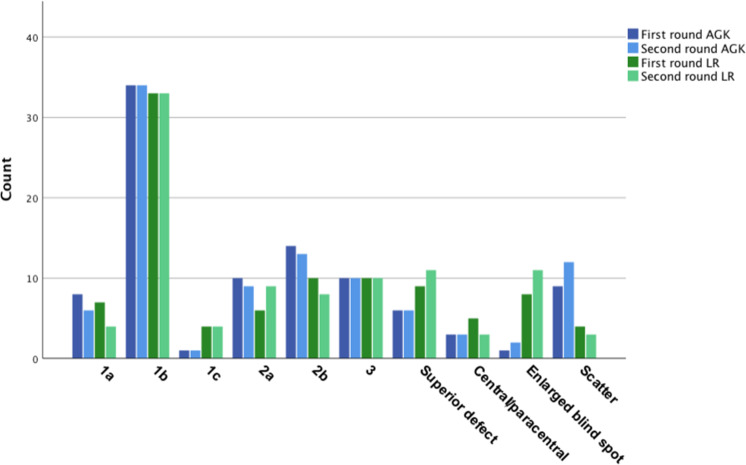
Frequency of the categories according to FT classification. The frequency of the ten categories in the Freitag–Tanking classification across the two readers (blue: AGK, green: LR) and two readings (darker shade: first round, lighter shade: second round) (color figure online).

Figure 1 shows the frequency of the ten categories across the two readers (blue: AGK, green: LR) and across two readings (darker shade: first round, lighter shade: second round).

Reader 1’s (LR) first reading (round 1) showed stage 1b to be the most common VF defect with 34.4% (34 eyes, 17 patients), followed by stage 2b and stage 3 defects both being 10.4% (both 10 eyes; 4 patients for stage 2b and 6 patients for stage 3), and superior defects with 9.4% (9 eyes, 7 patients) (Fig. 1).

Reader 2’s (AGK) first reading (round 1) similarly found stage 1b to be the most common VF defect with 35.4% (34 eyes, 18 patients), followed by stage 2b with 14.6% (14 eyes, 6 patients), and with stage 2a and stage 3 (each accounting for 10.4%, 10 eyes, 5/6 patients) trailing closely behind.

### Interreader agreement

The overall agreement between the two readers (1: LR, 2: AGK) was substantial (69.8% agreement, kappa 0.635 (95% CI [0.525–0.745])) for the first reading (round 1) and only slightly lower (66.7% agreement, kappa 0.598 (95% CI [0.488–0.708])) for the second reading (round 2).

The level of agreement varied across the different stages of the VF defects. At the first reading (round 1), the interreader agreement reached its highest level in stage 3 (100%), while remaining at substantial levels for stages 2a (60%), 2b (60%), and 1 (58.5%) as well as for superior defects (66.7%). The agreement was considerably lower for central/paracentral defects (33.3%), scatter defects (18.2%) and enlarged blind spot (12.5%).

At the second reading, the relative ordering of agreement with respect to stages of the VF defects changed little. The highest level of interreader agreement occurred for stage 3 (100%), followed by substantial agreement around stages 1 (64.2%), 1a (66.7%), 1b (64.3%), 1c (80%), stage 2a (50%), 2b (46.7%), and on superior defects (66.7%). The agreement on central/paracentral defects was higher compared to the first reading (50%), while that around scatter defects (27.3%) and enlarged blind spot (12.5%) remained similarly low.

The consistently perfect interreader agreement on stage 3 indicates that the FT classification has identified a particularly crisp definition of this particular category. Conversely, the recurring low levels of interreader agreement on scatter defects and enlarged blind spot suggest a relatively fuzzy definition of these stages in the FT classification.

### Intrareader reproducibility

Intrareader reproducibility (based on comparisons of readings at least a month apart) was substantial for reader 1 (LR) (78.1% agreement, kappa 0.736 (95% CI [0.638–0.834])) and near perfect for reader 2 (AGK) (90.6% agreement, kappa 0.885 (95% CI [0.814–0.956])).

### Mean deviation (MD) of the visual field

**Fig. 2:**
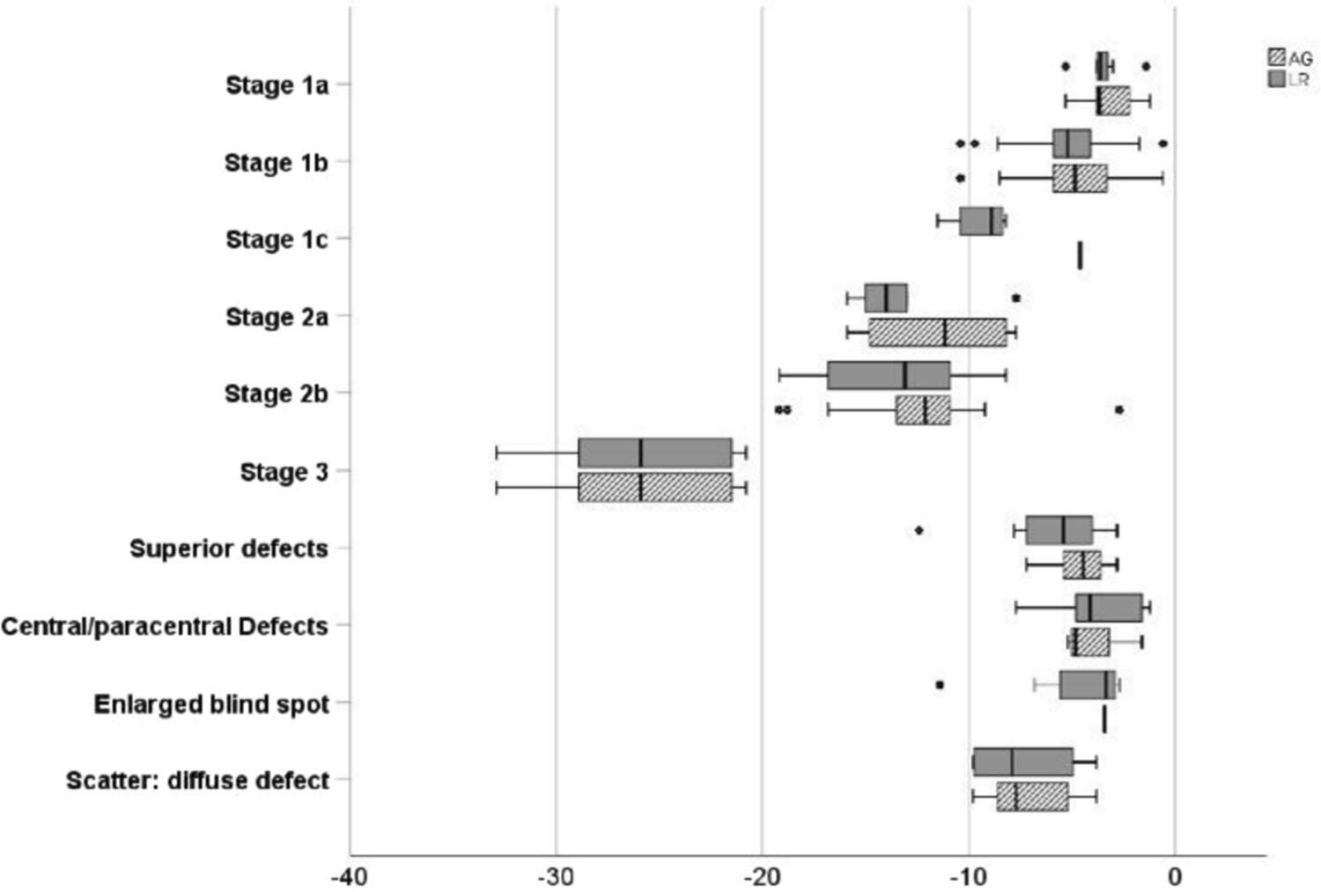
The distribution of MD of the visual field examination according to the stages of FT classification. The distribution of mean deviation (MD) across the ten categories in the Freitag–Tanking classification and across two readers (white with grey lines: AGK, solid grey: LR).

We computed the mean deviation (MD) for each category and for each reader during the first round of readings. (The results for the second round are similar and available upon request). Figure 2 shows box plots depicting the distribution of MD values across the stages and readers. The mean MD was lowest for stage 3 for both readers (AGK: −25.65, range [−32.9, −20.8] and LR: −25.65, range [−32.9, −20.8]). The mean MD was moderately low for both readers for stage 2a (AGK: −11.33, range [−15.9, −7.7] and LR: −13.27, range [−15.9, −7.7]) and stage 2b (AGK: −12.44, range [−19.2, −2.7] and LR: −13.46, range [−19.2, −8.2]).

### BCVA LogMAR

**Fig. 3:**
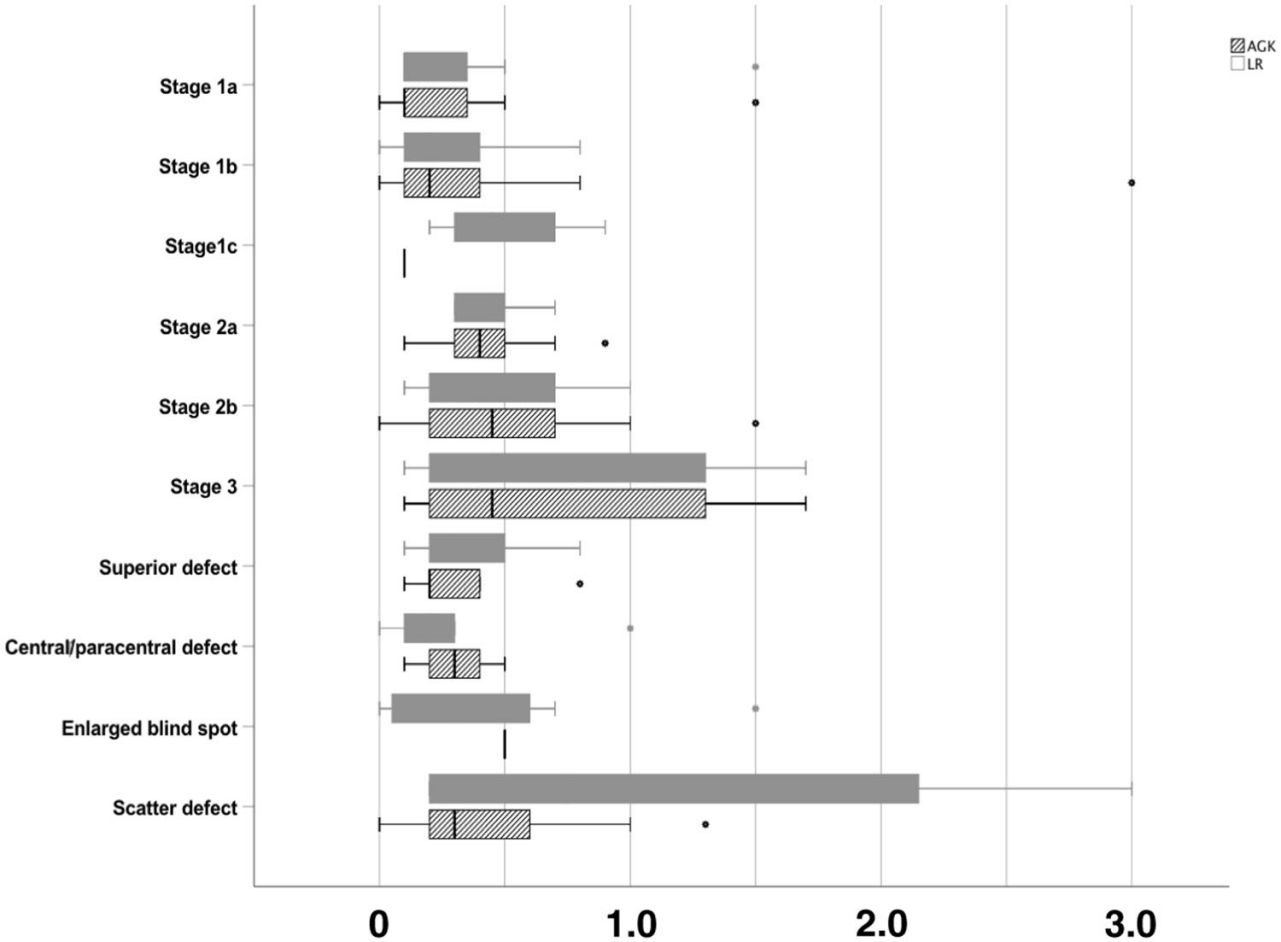
The distribution of BCVA (LogMAR) according to the stages of FT classification. The distribution of BCVA across the ten categories in the Freitag–Tanking classification and across two readers (white with grey lines: AGK, solid grey: LR).

Finally, we computed BCVA for each stage of the FT classification and for each reader in order to see correlation of the stages of the VF defects to the BCVA. Figure 3 shows box plots of the BCVA values by stage and reader. The results show the distribution of BCVA within each stage to be similar across the two readers for all stages except the scatter defects (subcategory of Stage X in FT) where the cases classified into this stage by reader 1 (LR) have a wider distribution of BCVA compared to those put into this stage by reader 2 (AGK).

Table 1 shows a summary of the clinical findings of the patients.

**Table 1:** Demographic and clinical data of the patients with TED-CON.

Characteristic	*N* (%)/Mean (range)
All	51 patients (96 eyes)
Patient demographic data	
Age	58.3 ± 10.3 years (range 34–79)
Sex	
Male	16 (31.4%)
Female	35 (68.6%)
Smoking history	
Current	23 (45.1%)
Ex-smoker	8 (15.7%)
None	17 (33.3%)
Missing	3 (5.9%)
TSH-R	18.7 (0.7–110.0)
TSH (µU/mL)	3.4 (0–34.0)
Clinical data for all included eyes	
BCVA (LogMAR)	0.42 LogMAR
CAS	5.6 (range: 1–8)
Intraocular pressure (mmHG)	18.2 mmHG (range:12–32)
Hertel Exopthalmometer (mm)	22.1 (range: 14–28)
RAPD	
Negative	77 (80.2%)
Positive	17 (17.7%)
Missing	2 (2.1%)
Optic disc oedema	
Yes	22 (22.9%)
No	69 (71.9%)
Missing	5 (5.2%)
Protan	
Normal	28 eyes (29.2%)
Pathological	55 eyes (57.3%)
Missing	13 eyes (13.5%)
Tritan	
Normal	1 eye (1%)
Pathological	82 eyes (85.4%)
Missing	13 (13.5%)

*TSH-R* TSH receptor autoantibodies, *TSH* Thyroid-stimulating hormone, *BCVA* Best-corrected visual acuity, *CAS* Clinical activity score, *RAPD* Relative afferent pupillary defect

## Discussion

Optic nerve compression in patients with TED is a rare complication, requiring urgent treatment and affecting 4–8% of patients with Graves orbitopathy (GO) [8, 9]. Until recently, lack of standardized diagnostic criteria for TED-CON patients has delayed timely diagnosis and treatment, and presented a persistent challenge in daily clinical life, even for the most experienced oculoplastic surgeons.

Many publications to date have described characteristics of TED-CON patients. McKeag et al., for example, examined 47 patients with suspected CON to identify the clinical features of the condition. Of the patients that were eventually diagnosed with definitive or equivocal TED-CON, 71% showed VF defects, compared to 13% of the patients who ended up not having the condition. The authors also noted optic disc swelling, impaired colour vision and radiological evidence of apical optic nerve compression as clinical signs confirming the diagnosis [10].

Recent findings have challenged the use of certain clinical findings for diagnosing TED-CON. For example, changes in BCVA were once considered a key criterion to diagnose this condition, but today, we recognize the presence of TED-CON cases with a good BCVA. One particular study shows 76% of the cases with CON to have a BCVA of 20/40 or better [11].

As a result, today, there is a pressing need to identify the clinical features of TED-CON in order to diagnose it accurately and prior to the deterioration of visual acuity. This need is underlined by our study, which finds that 45.8% eyes (44 out of 96) with TED-CON diagnosis present a BCVA LogMAR ≤0.2. Put differently, nearly half of the cases with the condition have a good BCVA. This pattern indicates the urgency of identifying criteria for detecting TED-CON in a timely manner and for preventing potential harm to visual acuity.

BCVA is not the only benchmark for diagnosing TED-CON. Indeed, researchers and clinicians have relied on other criteria, such as neuro-ophthalmological findings (e.g., RAPD and optic disc swelling). Due to the bilateral involvement of the TED-CON, however, the RAPD does not seem reliable. Indeed, this criterion is positive only in 45% of the diagnosed cases [10]. Similarly, the frequency of the optic disc swelling varies from 20 to 56% in TED-CON cases, and thus, is far from allowing a definitive diagnosis [10, 11].

Recent years have witnessed a number of important publications on the diagnosis, monitoring, and treatment of TED-CON [5, 12, 13]. There has been a significant effort on the improvement of the diagnostic findings in TED-CON patients pointing out the importance of the additional diagnostic features such as changes in colour vision and VF, even in subclinical cases [5, 14]. FT’s work, in particular, has made a key contribution with its classification of VF defects in describing the progression of the disease. This work offers two important advantages in daily clinical life. First, the classification alerts us to the very first changes in the inferior part of the VF, which can be an early sign of the optic nerve compression that is not reflected in BCVA. Second, the classification allows us to understand and categorize the progression of the disease in a standardized way.

In this study, our goal was to apply the FT classification to VF examinations from 51 patients (96 eyes) at the time of TED-CON diagnosis. We also wanted to evaluate this classification in terms of its reliability across examiners, and its reproducibility by the same examiner at different times. To that end, we de-identified patient records, randomized their ordering, and asked two examiners to classify VF defects independently. After a month of this initial reading, we re-numbered patient records (while still keeping them anonymous) and asked each reader to re-do the classification. By analysing the resulting data, we assessed both interreader agreement, and intrareader reproducibility of this important classification scheme for TED-CON.

We found that a wide distribution of our patients (eyes) across the ten stages of VF defects identified in the FT classification. The most frequent VF defects at the time of the diagnosis involved, what FT call, stage 1b (large inferior paracentral hemifield defect). These defects accounted for 34.4% of the eyes in reader 1’s classification, and 35.4% of the eyes in reader 2’s classification. The second most frequent defects were stage 2b (inferior altitudinal plus superior arcuate defect), making up 10.4% of cases for reader 1 and 14.6% cases for reader 2. The third most frequent defects belonged to stage 3 (total loss), including 10.4% cases for both readers.

We concluded that the FT classification is easy to understand and apply in daily clinical life. Similar to FT, we observed in our data the inferior defects to be the most common ones in TED-CON patients, and thus, confirmed the precision of the classification for detection this particular pathology. And, although we did not have a longitudinal design like FT, the fact that we observed similar frequencies of defects with observations at a single point in time underlines the usefulness of the classification even with limited data.

Comparing independent categorizations of two readers, we found out that the classification is most fuzzy when it comes to describing defects of the enlarged blind spot. This is where the greatest discrepancy in our categorizations occurred. We observed similar uncertainty in other defects subsumed under FT’s Stage X (central/paracentral, enlarged blind spot, or scatter). Our interreader agreement remained in the 12.5–50% range for this category, while it was around or over 60% for the other categories.

A main advantage of the FT classification—its ease of use—became evident in our assessment of intrareader reproducibility. The agreement between the readings at two time points was near perfect for one reader (90.6% agreement, kappa 0.885 (95% CI [0.814–0.956])) and only slightly lower for the other reader (78.1% agreement, kappa 0.736 95% CI [0.638–0.834]).

The FT classification of the VF abnormalities in TED-CON patients is a useful, easy-to-use and easy-to-learn method with a near-perfect intraobserver reproducibility and substantial interreader agreement. This classification is especially precise in identifying defects in the inferior region of the VF, ensuring substantial interreader agreement in our case (58.5–100%). It is worth noting that the classification lead to perfect interreader agreement for stage 3 defects (where the MD value suggested a cut-off >20 dB).

The COVID-19 pandemic has imposed binding constraints on our diagnostic resources and highlighted the importance of alternative methods for detecting hard-to-diagnose conditions, such as TED-CON. We point out two alternative strategies for detecting TED-CON (other than the tests we have considered in this study). The first strategy is based on Neigel et al.’s [9] work, which identified the greying of vision (or desaturation of colours) as a chief symptom in TED-CON patients. But, crucially, patients mentioned this symptom only when directly asked about it. Therefore, when faced with restrictions on diagnostic tools available to them, clinicians can simply ask their patients about this particular symptom when they suspect TED-CON as a possible condition. The second strategy for diagnosing TED-CON, one that would shorten the examination time and provide a more objective measure, is to use optical coherence tomography (OCT) imaging. Zhang et al. [15], for example, detected a decrease in vessel density in the peripapillary area in TED-CON patients with optical coherence angiography. Similarly, Park et al. [16] found significant difference in inferior peripapillary retinal nerve fibre layer between acute and chronic TED-CON patients, as well as between TED-CON patients and a control group, using OCT [16]. These findings suggest the potential of imaging tools for detecting TED-CON, but more future work is needed to fully evaluate the usefulness of these alternative tools. We do not feel VF screening of all TED patients for TED-CON is realistic, particularly in the post COVID healthcare landscape. We recommend VF testing only patients with suspected TED-CON to confirm the diagnosis and to monitor for progression.

It is worth emphasizing that BCVA would not have been a good predictor of TED-CON in our data. We had 44 eyes (45.8%) with a BCVA LogMAR ≤ 0.2, that is, half of cases had a good VA. Even more striking is the fact that about a fourth of our cases (22 eyes) had a BCVA LogMAR ≤ 0.1, indicating minimal-to-no worsening in BCVA. This pattern points to the need for performing other tests, such as VF examination, in suspected TED-CON patients as soon as possible. Similar to FT, our findings show the most frequent VF defect in TED-CON patients to be stage 1b defects. Therefore, clinicians should remain alert to small defects in inferior region (stage 1a/1b) of the VF examination, which might indicate potential TED-CON, even in patients with minimal-to-no worsening of the BCVA.

## Summary

### What was known before

- According to the latest publication of Freitag and Tanking on visual field (VF) defects of the patients with Thyroid Eye Disease-Compressive Optic Neuropathy (TED-CON), the VF defects are most often in the inferior region of the VF.
- The new classification system allows us to understand and categorise the progression of the TED-CON disease in a standardised way.

### What this study adds

- The FT classification of the VF abnormalities in TED-CON patients is a useful, easy-to-use and easy-to-learn method with substantial interreader agreement and near-perfect to substantial intraobserver reproducibility.
- In addition to evaluating the classification, we considered the relationship between VF defects and best-corrected visual acuity (BCVA) in our patients. We found good visual acuity indicated by BCVA LogMAR ≤0.2, in nearly half of the cases (44 eyes, 45.8%). More strikingly, we observed near perfect visual acuity (BCVA LogMAR ≤0.1) in about a quarter of the cases (22 eyes, 22.9%) with TED-CON diagnosis.
- Clinicians should be alert to VF defects in the inferior region (stage 1a/1b in the FT classification) even in patients showing minimal-to-no worsening of the BCVA.
